# Combined arthroscopic rotator cuff repair with mesenchymal stem cell augmentation shows similar functional outcomes but a higher structural integrity rate compared with isolated repair: a meta-analysis of comparative studies

**DOI:** 10.1016/j.jseint.2025.03.017

**Published:** 2025-04-23

**Authors:** Erminia Cofano, Roberto Minici, Giovanna Spina, Domenico Laganà, Olimpio Galasso, Giorgio Gasparini, Italian Orthopaedic Research Society, Michele Mercurio

**Affiliations:** a Department of Orthopaedic and Trauma Surgery, “Magna Græcia” University, “Renato Dulbecco” University Hospital, Catanzaro, Italy; b Radiology Unit, Department of Experimental and Clinical Medicine, “Magna Græcia” University, “Renato Dulbecco” University Hospital, Catanzaro, Italy; c Department of Medicine, Surgery and Dentistry, University of Salerno, Salerno, Italy; d Research Center on Musculoskeletal Health, MusculoSkeletalHealth@UMG, Magna Graecia University, Catanzaro, Italy; e Italian Orthopaedic Research Society, Roma, Italy

**Keywords:** Shoulder, Arthroscopic RCR, Mesenchymal stem cells, Functional outcomes, Complications, Retear

## Abstract

**Background:**

Arthroscopic rotator cuff repair (RCR) has guaranteed satisfactory outcomes but remains associated with a significant rate of tendon-bone healing failure. Mesenchymal stem cells (MSCs) have been tested as a promising cell-based therapy for rotator cuff tear (RCT). MSC augmentation has been proposed as a therapy associated with surgical repair, potentially enhancing the overall surgical outcomes for patients with RCTs. The aim of this meta-analysis was to compare functional and structural outcomes between arthroscopic RCR combined with MSC augmentation vs. isolated RCR for RCT.

**Methods:**

The PubMed, MEDLINE, Scopus, and Cochrane Central databases were used to search keywords, and 5 studies were included. The first author, journal name, year of publication, study design, patient demographics, type of surgery, and follow-up period were recorded. The data extracted for quantitative analysis included the visual analog scale, University of California at Los Angeles score, flexion, external rotation, and retear. Random and fixed effect models were used for the meta-analysis of pooled mean differences and odds ratios.

**Results:**

A total of 415 patients were identified, 203 of whom underwent combined RCR and MSC augmentation, and 212 underwent isolated RCR. The mean ages were 62.2 ± 6.2 and 61.6 ± 5.7 years in the combined RCR and MSC injection and isolated RCR groups, respectively. The mean follow-up was 17.5 ± 8.7 and 17.6 ± 8 months. Comparable postoperative visual analog scale score (*P* = .59), flexion (*P* = .68), external rotation (*P* = .42), and University of California at Los Angeles score (*P* = .92) were found between the groups. Significantly higher rotator cuff retear rate was found in the isolated RCR group (17.7% and 35% for the RCR and MSC injection and isolated RCR groups, respectively; odds ratio = −0.19, 95% confidence interval [−0.34, −0.04], *P* = .01).

**Conclusion:**

Arthroscopic surgical repair combined with MSC augmentation reported better structural outcomes compared to isolated surgical repair for RCT. Pain and functional outcomes were similar between the two groups.

Rotator cuff tears (RCTs) are prevalent injuries that result in shoulder pain and functional impairment, often requiring surgical intervention.^28^ Although arthroscopic rotator cuff repair (RCR) has gained widespread acceptance for delivering satisfactory outcomes, it remains associated with a significant rate of tendon-bone healing failure.^51^ This is primarily due to challenges in re-establishing the complex transitional tissue between the tendon and the bone.^3^ Literature indicates that around 20% of surgically repaired rotator cuffs fail to heal adequately^13^ irrespective of the surgical techniques used.^29^ Consequently, various strategies have been explored to enhance the mechanical support of the repair construct and to biologically augment tendon-to-bone healing, aiming to reduce the retear rate postsurgery.^22^^,^^45^

Interestingly, rotator cuff retear is a multifactorial phenomenon in which biological causes prevail over mechanical ones.^29^ Mesenchymal stem cells (MSCs) have been tested as a promising cell-based therapy for RCT.^8^^,^^15^^,^^16^^,^^19^^,^^58^ MSCs facilitate the regeneration of the bone-tendon interface by proliferating and differentiating, exerting paracrine effects on nearby tissues, and stimulating collagen production via cytokines and growth factors. Hence, MSC augmentation has been proposed as a therapy associated with surgical repair, potentially enhancing the overall surgical outcomes for patients with RCTs.

Although human trials on MSC are limited, they indicate a potential for reducing retear rates and improving postrepair outcomes.^19^^,^^22^^,^^26^ However, the inconsistent results from randomized controlled trials create uncertainty among clinicians and patients about the true clinical benefits of this therapy.^5^^,^^46^ Our study aimed to contribute to the current knowledge by synthesizing existing evidence and aggregating published data. We conducted a systematic review and meta-analysis of functional and structural outcomes reported in comparative studies evaluating arthroscopic RCR combined with MSC augmentation vs. isolated RCR for RCT.

## Materials and methods

### Search strategy

A systematic review of the published literature was conducted and reported according to the Preferred Reporting Items for Systematic Reviews and Meta-Analyses statement.^40^ The PubMed, MEDLINE, Scopus, and Cochrane Central databases were searched in September 2023 with no lower date limit. The terms “mesenchymal stem cell”, “shoulder”, “arthroscopy”, “rotator cuff repair”, “outcome”, and “results” were used in different combinations to retrieve relevant articles. The articles were selected based on the following PICO model^50^: (P) patients who underwent arthroscopic RCR; (I) MSC augmentation; (C) patients who did not undergo additional MSC augmentation; and (O) patients assessed for functional and structural outcomes.

Two authors (G.S. and E.C.) independently conducted all the searches and screened the titles and abstracts to identify articles for inclusion. If a study could not be excluded based on the title and abstract, both reviewers reviewed the full text to reach a consensus on the inclusion or exclusion of the study by contacting a third author (M.M.) in case of major discrepancies. The reference list of each included article and the available gray literature at our institution were screened for the inclusion of potential additional articles.

### Inclusion criteria and study selection

The following inclusion criteria were applied during the title, abstract, and full-text screenings: (1) observational studies including case‒control studies, cohort studies, and experimental studies including randomized controlled trials; (2) reported comparative outcomes of combined arthroscopic RCR and MSC augmentation and isolated arthroscopic RCR; (3) reported >10 surgically treated cases; and (4) written in English. The exclusion criteria were as follows: (1) studies involving isolated MSC augmentation; (2) injection of platelet-rich plasma (PRP) and other growth factors; (3) tendon allograft or autograft transplantation; and (4) revision treatment of RCR. Other reviews, case reports, cadaveric or biomechanical studies, technical notes, editorials, letters to the editor, and expert opinions were excluded from the analysis but considered for the discussion section.

### Data extraction and quality assessment

Two authors (G.S. and M.M.) performed comprehensive data extraction from the included articles. The first author, journal name, year of publication, study design, patient demographics, type of surgery, and follow-up period were recorded. The data extracted for quantitative analysis included the visual analog scale, University of California at Los Angeles score,^44^ flexion, external rotation, and retear. A methodological quality assessment was independently conducted by 3 authors (G.S., E.C., and M.M.) with the modified Newcastle–Ottawa Quality Assessment Scale^59^ (Table I). Substantial interobserver agreement (Cohen kappa coefficients ranging between 0.57 and 0.72) was reported.

**Table I:** Quality assessment of included studies according to the Modified Newcastle–Ottawa scale.

Study author (yr)	Criteria								Total	Quality
	**1**	**2**	**3**	**4**	**5**	**6**	**7**	**8**		
Kim et al (2020)	1	1	1	1	1	2	1	1	9	High
Kim et al (2017)	1	1	1	1	1	2	1	1	9	High
Hernigou et al (2014)	1	1	1	1	1	1	1	1	8	High
Mazzocca et al (2010)	1	1	1	1	1	1	1	1	8	High
Taniguchi et al (2015)	1	1	1	1	1	1	1	1	8	High

Based on the total score, quality was classified as “low” (0-3), “moderate” (4-6) and “high” (7-9). Criterion number (in bold): 1, representativeness of the exposed cohort; 2, selection of the nonexposed cohort; 3, ascertainment of exposure; 4, demonstration that outcome of interest was not present at start of study; 5, comparability of cohorts on the basis of the design or analysis; 6, assessment of outcome; 7, was follow-up long enough for outcomes to occur?; 8, adequacy of follow up of cohorts. Each study was awarded a maximum of one or two points for each numbered item within categories, based on the Modified Newcastle–Ottawa scale rules.

### Data synthesis

All data were reported with 1-decimal accuracy. The mean, standard deviation, and range were noted for the continuous variables, and counts were noted for the categorical variables. The functional outcomes and complications were entered into a meta-analysis of pooled mean differences (MDs) and odds ratios, respectively. The Mantel–Haenszel method was adopted according to the Cochrane statistical methods group. Random or fixed-effect models were used based on between-trial heterogeneity as calculated by the I^2^ statistics; in particular, random-effect models were used when considerable heterogeneity (I^2^ > 50%) was noted, unless the between-studies variance (σ^2^) was poor, in which case fixed-effect models were used despite the heterogeneity found.^38^ Review Manager (RevMan 5.3; Cochrane Collaboration, Nordic Cochrane Center, Copenhagen, Denmark) was used for the statistical calculations; a *P* value < .05 was considered indicative of statistical significance.

## Results

**Figure 1:**
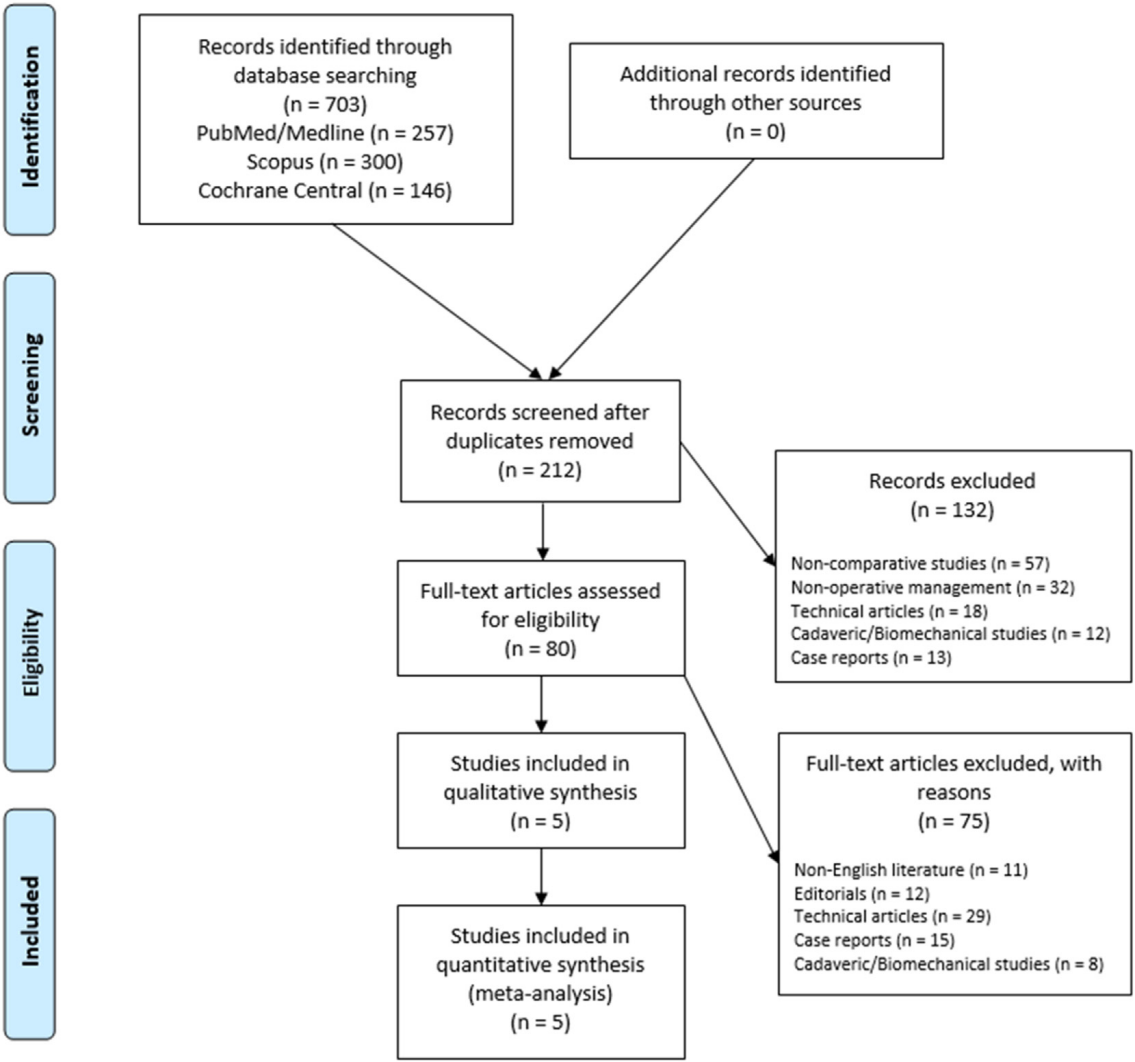
Preferred Reporting Items for Systematic Review and Meta-Analysis (PRISMA) flowchart for the searching and identification of included studies. Source: Moher et al. For more information, visit www.prisma-statement.org.

A total of 703 relevant articles were identified through the initial search, 212 abstracts were screened, and 80 full-text articles were assessed for eligibility based on our inclusion criteria, resulting in 5 comparative studies that were eligible for the meta-analysis (Fig. 1).

### Epidemiological data

The included studies were published from 2010 to 2020. Two studies were conducted in the Republic of Korea,^25^^,^^26^ one in France,^19^ one in Japan,^55^ and one in the United States.^32^

A total of 415 patients were initially identified, 203 of whom underwent combined RCR and MSC augmentation and 212 of whom underwent isolated RCR. There were 101 (49.7%) and 102 (48.1%) female patients in the 2 groups, respectively.

The mean ages were 62.2 ± 6.2 and 61.6 ± 5.7 years in the combined RCR and MSC augmentation and isolated RCR groups, respectively. The mean follow-up was reported in 3 studies,^26^^,^^32^^,^^55^ and it was 17.5 ± 8.7 and 17.6 ± 8 in the two groups, respectively (Table II).

**Table II:** Characteristics of included studies.

Author	Journal	Year of publication	Level of evidence	Group	Patients demographic							Functional outcomes							
					Number of patients (N)	Sex (N)		Age (yr)		FU (mo)		VAS postoperative		UCLA postoperative		Flexion postopertive		ER postoperative	
						M	F	Mean	SD	Mean	SD	Mean	SD	Mean	SD	Mean	SD	Mean	SD
Kim et al	The American Journal of Sports Medicine	2020	IV	RCR + MSC	56	26	30	64.6	6	24	NA	1.2	1.4	28.5	6.4	145	18	52	11
				RCR	42	23	19	64.2	5.5	24	NA	1.3	1.4	27.4	6.7	145	30	50	11
Kim et al	The American Journal of Sports Medicine	2017	III	RCR + MSC	35	15	20	59.2	3.4	28.3	3.8	0.4	0.6	29.8	5.1	155.2	25	65.5	23.4
				RCR	35	13	22	57.6	2.9	28.8	4.2	0.3	0.5	30.5	4.8	156.4	23.2	67.2	21.5
Hernigou et al	Inernational Orthopaedics	2014	IV	RCR + MSC	45	28	25	NA	NA	NA	NA	NA	NA	NA	NA	NA	NA	NA	NA
				RCR	45	28	25	61	5.5	NA	NA	NA	NA	NA	NA	NA	NA	NA	NA
Taniguchi et al	Journal of Shoulder and Elbow Surgery	2015	III	RCR + MSC	44	22	15	64.7	1.4	12.6	0.2	NA	NA	NA	NA	NA	NA	NA	NA
				RCR	67	42	25	64.3	1.1	14.4	0.5	NA	NA	NA	NA	NA	NA	NA	NA
Mazzocca et al	The American Journal of Sports Medicine	2010	III	RCR + MSC	23	12	11	56	8.7	10.6	6.7	NA	NA	NA	NA	163	31	65	20.4
				RCR	23	12	11	56	8.9	10	6.2	NA	NA	NA	NA	145.7	41.4	62.5	17.1

*FU*, follow-up; *VAS*, visual analog scale; *UCLA*, University of California at Los Angeles; *ER*, external rotation; *SD*, standard deviation; *RCR*, rotator cuff repair; *MSC*, mesenchymal stem cell; *NA*, not available.

In 4 studies, the MSCs were derived from bone-marrow stimulation,^19^^,^^25^^,^^32^^,^^55^ and in 1 study, the MSCs were derived from adipose tissue.^26^

### Functional outcomes

**Figure 2:**
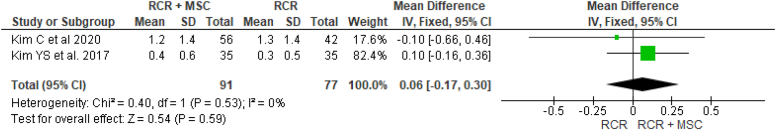
Comparison of the postoperative VAS score between RCR with MSC and isolated RCR groups: forest plot of effect sizes. *VAS*, visual analog scale; *RCR*, rotator cuff repair; *MSC*, mesenchymal stem cell; *CI*, confidence interval; *SD*, standard deviation.

Two studies^25^^,^^26^ investigated the preoperative and postoperative visual analog scale score in 91 patients in the RCR and MSC augmentation group and 77 patients in the isolated RCR group. No statistically significant difference was found between the two group preoperatively (4.8 ± 2.6 and 4.6 ± 2.5 for the RCR and MSC and isolated RCR groups, respectively, MD = 0.09, 95% confidence interval [CI; −0.58, 0.77], *P* = .79) (Supplementary Material 1), and postoperatively (0.9 ± 1.2 and 0.8 ± 1.2 for the RCR and MSC and isolated RCR groups, respectively, MD = 0.06, 95% CI [−0.17, 0.30], *P* = .59) (Fig. 2).

**Figure 3:**
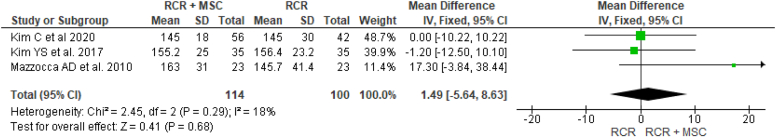
Comparison of the postoperative flexion between RCR with MSC and isolated RCR groups: forest plot of effect sizes. *RCR*, rotator cuff repair; *MSC*, mesenchymal stem cell; *CI*, confidence interval; *SD*, standard deviation.

**Figure 4:**
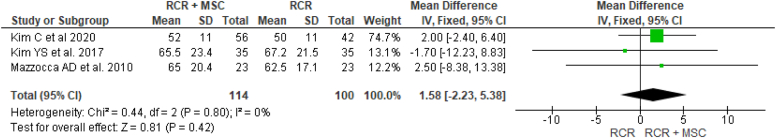
Comparison of the postoperative external rotation between RCR with MSC and RCR groups: forest plot of effect sizes. *RCR*, rotator cuff repair; *MSC*, mesenchymal stem cell; *CI*, confidence interval; *SD*, standard deviation.

Two studies^25^^,^^26^ reported preoperative flexion and external rotation in 91 patients in the RCR and MSC augmentation group and 77 patients in the isolated RCR group, and no difference was found between the groups. For preoperative flexion 135.9 ± 25.2 and 135.2 ± 26.8 for the combined RCR and MSC augmentation and isolated RCR groups, respectively, MD = 2.43, 95% CI (−4.76, 9.62), *P* = .51; for external rotation 46.6 ± 16.8 and 46.3 ± 17.3 for the combined RCR and MSC augmentation and isolated RCR groups, respectively, MD = 1.79, 95% CI (−2.83, 6.40), *P* = .45 (Supplementary Material 2 and Supplementary Material 3). Three studies^25^^,^^26^^,^^32^ reported the postoperative flexion and the postoperative external rotation in 114 patients in the combined RCR and MSC augmentation group and 100 patients in the isolated RCR group, and no difference was found between the groups (151.8 ± 24.4 and 149.1 ± 31 for the combined RCR and MSC augmentation and isolated RCR groups, respectively, MD = 1.49, 95% CI [−5.64, 8.63], *P* = .68 for flexion; 58.8 ± 18.7 and 57.8 ± 18.6 for the combined RCR and MSC augmentation and isolated RCR groups, respectively, MD = 1.58, 95% CI [−2.23, 5.38], *P* = .42 for the external rotation) (Figs. 3 and 4).

**Figure 5:**
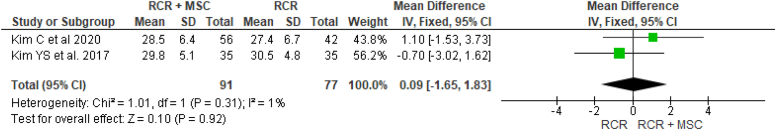
Comparison of the postoperative UCLA score between RCR with MSC and RCR groups: forest plot of effect sizes. *UCLA*, University of California at Los Angeles; *RCR*, rotator cuff repair; *MSC*, mesenchymal stem cell; *CI*, confidence interval; *SD*, standard deviation.

Two studies^25^^,^^26^ reported the preoperative and postoperative University of California at Los Angeles score in 91 patients in the RCR and MSC augmentation group and 77 patients in the isolated RCR group, and no difference was found between the groups preoperatively (19.7 ± 7.5 and 11.3 ± 24.5 for the combined RCR and MSC augmentation and isolated RCR groups, respectively, MD = 0.23, 95% CI [−1.40, 1.87], *P* = .78) (Supplementary Material 4) and postoperatively (29 ± 6 and 28.8 ± 6.1 for the combined microfracture and MSC augmentation and isolated microfracture groups, respectively, (MD = 0.09, 95% CI [−1.65, 1.83], *P* = .92) (Fig. 5).

### Structural outcomes

**Figure 6:**
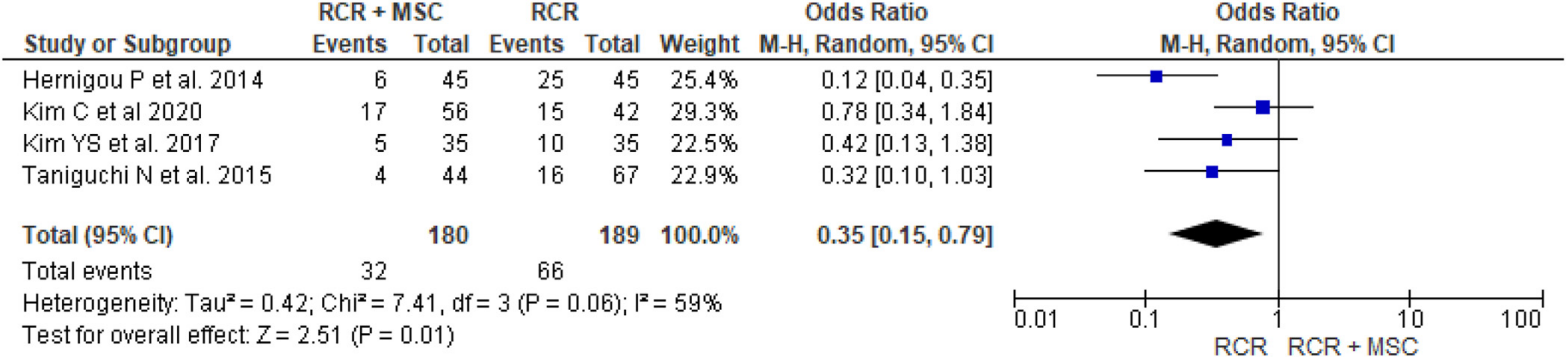
Comparison of the retear rate between RCR with MSC and isolated RCR groups: forest plot of effect sizes. *RCR*, rotator cuff repair; *MSC*, mesenchymal stem cell; *CI*, confidence interval.

Four studies^19^^,^^25^^,^^26^^,^^55^ investigated the rotator cuff retear rate in 180 patients in the RCR and MSC augmentation group and 189 patients in the isolated RCR group. A significantly higher rotator cuff retear rate was found in the isolated RCR group (17.7% and 35% for the RCR and MSC augmentation and isolated RCR groups, respectively; odds ratio = 0.35, 95% CI [0.15, 0.79], *P* = .01) (Fig. 6).

## Discussion

The key insight from this meta-analysis was that patients who underwent RCR combined with MSC augmentation have a significantly lower retear rate than those who underwent isolated RCR. Postoperative pain and functional outcomes were similar. The two groups analyzed were well-matched in terms of the number of patients, gender distribution, average age, and mean follow-up.

Although traditionally viewed as a mechanical failure, more recent data have advocated the pivotal role of biological changes in RCT. This is particularly evident in the higher incidence of RCT with advancing age.^13^ Similarly, the question of why early structural failure of RCR occurs despite advanced biomechanical repair techniques can often be attributed to biological factors. Indeed, successful RCR requires both initial mechanical strength and adequate biological healing potential in the tendon and bone.^45^ Iannotti et al revealed that 94.7% of retears occurred within the first 6 months,^21^ suggesting that initial tendon-bone interface healing is often the “weak link”.

Exploring the theoretical basis of RCT provides fundamental clues toward a more complete understanding of the role of MSC augmentation during RCR. Several studies agreed that both extrinsic (eg, soft tissue or bony impingement) and intrinsic (eg, aging and vascular supply) factors contribute to tendon pathology.^30^^,^^31^ The critical vascular zone’s impact on tendon pathology is a key factor.^41^ Hypovascularity is both a contributor to and a result of^11^^,^^18^^,^^54^ degenerative lesions, thus fueling speculations about a spectrum continuum of the vascular contribution to tendon degeneration, tear, and retear after surgery.

After a RCT, the injury site undergoes a healing process involving the following 3 overlapping stages: inflammation, proliferation, and remodeling. During these stages, various growth factors such as basic fibroblast growth factor, bone morphogenetic proteins, transforming growth factor-beta, and vascular endothelial growth factor are released by cells to aid tissue repair and remodeling.^7^ Angiogenic factors promote the formation of a new vascular network to supply blood to the newly formed fibrous tissue.^9^^,^^18^ The remodeling phase aims to restore tendon stiffness and tensile strength to preinjury levels by modifying the extracellular matrix.^56^ This process can take up to 12 months, increasing the risk of retearing.

Kida et al discovered that creating drilled holes in the humeral footprint could encourage the migration of autologous bone marrow-derived MSCs (BMSCs) to the repair site, thereby enhancing tendon-bone healing and increasing the ultimate force-to-failure.^24^ In this context, MSC augmentation resulted in markedly elevated levels of aggrecan, type II collagen, and hyaline-like components in the repaired tissue as demonstrated by immunohistochemical analysis.^10^^,^^34^ MSCs play a crucial role in enhancing tendon-bone healing and improving biomechanical strength, promoting early fibrocartilage formation, increasing collagen diameter and alignment, boosting paracrine factors production including cytokines, chemokines, growth factors, and microRNAs.^4^^,^^16^^,^^17^^,^^24^ In addition, MSC enhance osteogenic differentiation and improve resistance to cell death, restoring bone mineral density at the enthesis^37^ and fostering better biomechanical properties in the newly formed bone.^17^

In a rabbit model of chronic RCT, researchers found that injecting MSC following RCR resulted in greater tendon load to failure, enhanced muscle stiffness, and improved tendon stress tolerance compared to surgical repair alone.^57^ According to human cohort study performed by Kim et al,^26^ the augmentation of surgical RCR with adipose-derived MSC significantly reduced retear rates as observed via magnetic resonance imaging.^14^^,^^39^ However, there were no postoperative clinical differences and these findings are consistent with our results. Cole et al revealed no significant differences in patient-reported pain and functional outcomes between the groups. Nevertheless, the augmented repair group exhibited a lower retear rate (18% compared to 57%) according to Sugaya classification on 1-year magnetic resonance imaging scans.^5^ Similarly, Taniguchi et al reported that the retear rate was significantly higher in the isolated RCR group (23.9%) than in the combined RCR with MSC group (9.1%).^55^ Consistent with the above mentioned reports, Hernigou et al conducted a study with a minimum 10-year follow-up demonstrating that patients who underwent biological augmentation with BMSC during arthroscopy experienced a significantly reduced rate of retear.^19^

Although the structural outcomes improved, the clinical outcomes showed no significant difference between the groups in the current meta-analysis. Interestingly, the discrepancy between structural and clinical outcomes could be due to the timing of evaluations. Studies have shown that most retears occur within the first 6 months postsurgery,^2^^,^^33^ making the mean follow-up periods appropriate for structural evaluation but likely insufficient to fully assess long-term clinical outcomes. Moreover, the results of the current study should be interpreted with caution; indeed, various authors, such as Kim et al,^26^ Sugaya et al,^53^ and Slabaugh et al,^52^ suggested that repair site integrity is linked to clinical improvement, therefore, a positive correlation between structural and clinical outcomes should be expected.

The economic impact of RCR is substantial; this includes both direct expenses and indirect costs such as lost productivity and worker’s compensation.^12^ The increasing incidence of revision RCR, driven by retears and poor patient outcomes,^35^ exacerbates the economic burden.^45^ Consequently, the current approaches to treating RCTs can be improved to address clinical, economic, and social challenge.^20^ A study by Samuelson et al^49^ conducted an analysis of cost-effectiveness of the use of other type of augmentation such as the PRP finding that this type of application is not cost-effective. The authors suggested that for the augmentation to be effective, the retear rate must be below 9.1%. In our study, the retear rate was 17.7% and 35% for the RCR and MSC augmentation and isolated RCR groups, respectively. In this light, it should be considered that the in situ delivery of MSC, especially with bone marrow stimulation used in 4 of 5 studies included in the current review, is a feasible surgical solution that limits operative costs and does not compromise postoperative recovery. Further studies are needed to assess the cost-effectiveness of MSC augmentation and compare it with other types of augmentation, such as the PRP, to assess their effectiveness and economic benefit.^1^

Several limitations of the current study should be noted. First, the choice of MSC sources is crucial in determining treatment efficacy, contributing to variability across studies. Different tissues used for MSC extraction differ in accessibility, cell concentration, and differentiation potential.^48^ BMSCs are well-studied^42^; however, bone marrow extraction is painful and can lead to complications.^43^ Thus, alternative sources are being explored to find those that are easier to extract, pose fewer risks, and provide high cell yields with consistent proliferation and differentiation abilities.^23^^,^^48^ Adipose-derived stem cells have been investigated, demonstrating the ease of accessibility and ability to modulate the microenvironment, exert anti-inflammatory properties, and differentiate into tenocytes,^6^^,^^27^^,^^47^ thus resulting in an augmentation potential similar to BMSC.^26^ Second, exogenous MSC can be transported to the repair site using a variety of carriers,^4^ thus introducing a potential confounding factor that has not been evaluated in our meta-analysis. Third, although radiological and functional outcomes are frequently assessed, it would be beneficial to have comparative studies that focus directly on histological outcomes. Fourth, we considered English studies only, potentially contributing to publication bias; moreover, although 4 recommended databases were used for the search, we cannot exclude that further articles could have been found using other databases.^36^ Nevertheless, major methodological strengths of this study are the comparative nature of the article inclusion strategy and the pooling of effect sizes to identify differences between the 2 fixation methods.

## Conclusion

Our meta-analysis revealed that the arthroscopic surgical repair combined with MSC augmentation reported better structural outcomes compared to isolated surgical repair for RCT. Pain and functional outcomes were similar between the 2 groups. These findings suggest that MSC augmentation helps reinforce the tendon-bone interface by addressing the biological limitations of the healing process.

## Disclaimers:

Funding: No funding was disclosed by the authors.

Conflicts of interest: Giorgio Gasparini is the president of the Italian Orthopaedic Research Society. Michele Mercurio is a member of the executive board of the Italian Orthopaedic Research Society. The authors, their immediate families, and any research foundation with which they are affiliated have not received any financial payments or other benefits from any commercial entity related to the subject of this article.
