# Supplementary material for: Combined arthroscopic rotator cuff repair with mesenchymal stem cell augmentation shows similar functional outcomes but a higher structural integrity rate compared with isolated repair: a meta-analysis of comparative studies
Source: JSES Int. 2025 Apr 23;9(4):1191–8. doi: 10.1016/j.jseint.2025.03.017 (PMC12435019; doi:10.1016/j.jseint.2025.03.017)
Supplement: Supplementary Material 2 [file mmc2.docx]

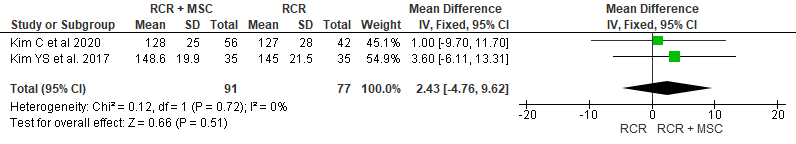


**SM2**. Comparison of the preoperative flexion between RCR with MSC and isolated RCR groups: forest plot of effect sizes
